# Editorial: Endothelial cells as innate immune cells

**DOI:** 10.3389/fimmu.2022.1035497

**Published:** 2022-10-04

**Authors:** Yifan Lu, Yu Sun, Keman Xu, Ying Shao, Fatma Saaoud, Nathaniel W. Snyder, Ling Yang, Jun Yu, Sheng Wu, Wenhui Hu, Jianxin Sun, Hong Wang, Xiaofeng Yang

**Affiliations:** ^1^ Centers of Cardiovascular Research, Temple University Lewis Katz School of Medicine, Philadelphia, PA, United States; ^2^ Metabolic Disease Research and Thrombosis Research Center, Department of Cardiovascular Sciences, Temple University Lewis Katz School of Medicine, Philadelphia, PA, United States; ^3^ Department of Medical Genetics and Molecular Biochemistry, Temple University Lewis Katz School of Medicine, Philadelphia, PA, United States; ^4^ Center for Translational Medicine, Department of Medicine, Simmel Medical College, Thomas Jefferson University, Philadelphia, PA, United States

**Keywords:** endothelial cells as immune cells, aorta as immune organ, secretome, vascular inflammation, trained immunity

## Introduction

With the great effort and support from the authors and editorial team, our Research Topic entitled “Endothelial cells as innate immune cells” in Frontiers in Immunology, Molecular Innate Immunity Section has achieved a great success and have attracted so far 6,775 views and numerous submissions. Endothelial cells are the innermost cell type lined along vessels in all the organs and tissues through hosts, indicating their anatomic and physiological roles in regulating vascular tone, preventing blood components from leaking and maintaining vascular functions (1). The traditional concept emphasized that innate immune cells are non-T cells and non-B cells, which migrate from blood circulation to inflammatory/injury/tumor sites. Our new concept states that immune responses and inflammation and tumorigenesis are highly coordinated processes. Regardless of migration and structural cell types, any cell types can be defined as innate immune cells, which are capable in autonomously sensing danger/pathogen associated molecular patterns (DAMPs/PAMPs) and providing signaling supports to these processes *via* cell membrane proteins (for example, clusters of differentiation, CDs) and secretomes/cytokines/chemokines/growth factors (2, 3). The pathological roles of endothelial cells in participating in various inflammations and immune responses were reported (3). However, immunological characters of endothelial cells have been poorly characterized due to the fact that endothelial cells have not been recognized as innate immune cells in the field. To fill in this significant knowledge gap, ten years ago based on more than ten major innate immune functional aspects that are shared by the prototypic innate immune cell type - macrophages and endothelial cells, we proposed a new concept that endothelial cells are innate immune cells (2, 4), which was further supported by our experimental data (5–13) analyses (Shao et al. , 15). The Molecular Innate Immunity field is continuously evolving, and we greatly appreciate the Molecular Innate Immunity section editors gave us this opportunity to organize this Research Topic and work with other investigators to explore this important topic further. Here, we are excited to see nearly five publications collected in our topic since 2021 (**Table 1**).

**Table 1:** Summary for 5 highlighted studies in Frontiers in Immunology: 2022.

Cell type/mouse model	Stimulation/condition	Finding	Reference
Endothelial cell	PAMPs/DAMPs	Endothelial cells and VSMCs have novel trained immunity *via* 6 types of secretomes	PMID: 35320939
Endothelial cell	ox-LDL,TNFa, IL1b	PHACTR1 mediates endothelial inflammation	Ma et al.
Endothelial cell	LPS, Ang II, virus	Upregulated RIG-I pathway mediates endothelial cell inflammatory response	PMID: 35865527
Endothelial cell	PAMPs/DAMPs	Endothelial cells should be considered macrophage-like gatekeepers	PMID: 35619719
Endothelial cell	SARS-CoV-2	Endothelial activation associated with COVID-19 is likely a result of inflammatory responses initiated by other cells.	PMID: 35837388

## Trained immunity is a novel mechanism for persistent hyperactivation and synergies between DAMPs/PAMPs

One of the significant progresses in the innate immunity field is the identification of innate immune memory or trained immunity (15–18). Ever since investigators found memory adaptive immune cells such as memory T cells and memory B cells, we have always been puzzled by two questions whether innate immune cells have memory functional status for challenged stimuli and whether innate immune cells have memory cell subtypes similar to that of adaptive immune cells. In response to danger/pathogen associated molecular patterns (DAMPs/PAMPs) and conditional DAMPs (19) derived from injury, lipid peroxidation (15), chronic kidney disease-related uremic toxins stimulation (20–22), hyperlipidemia, hyperglycemia (23), hyperhomocysteinemia (24–26), metabolic syndrome, hypertension, cigarette smoke, bacterial infections, and virus infections, inflammations take place in vasculature (27). However, two significant questions remain whether vascular innate immune cell types including endothelial cells and vascular smooth muscle cells (VSMCs) have any innate immune memory function to remember risk factor challenges; and whether innate immune cells response differently to the second stimulation after exposed to the first stimulation. Benefit from immunological, metabolic and epigenetic research progresses, new innate immune memory function or trained immunity has been identified. The inflammatory microenvironment and DAMPs/PAMPs can keep aortic endothelial cells, VSMCs, monocytes, macrophages and other innate immune cells persistent hyperactivation and will develop exacerbated immunologic response to the second stimulation (trained immunity) (16, 28, Zhong et al.). For current understanding, trained immunity (15, 16, Zhong et al.) is formed *via* metabolic reprogramming (29) and epigenetic memory (10, 30, 31). All the cell types in aorta participate in this process including but not limited to endothelial cells, VSMCs (21, 32), monocytes, macrophages, neutrophils, T cells, CD4^+^ regulatory T cells (Treg) (33–37) (Ni et al.; Shao et al.; Xu et al., Zhang et al.) and B cells. In this editorial we will discuss the most recent research and novel insight in endothelial cells (Shao et al.), macrophages (Barhoumi et al.; Dominguez et al.; Lai et al.; Li et al.; Zhang et al.) and neutrophil (Domer et al.; Perez-Figueroa et al.; Rydzynska et al.), which may contribute to the formation of trained immunity in aorta.

It has been reported that endothelial cell is an innate immune cell and can upregulated three major metabolic pathways including glycolysis pathway, mevalonate pathway and acetyl-Co-A synthesis to build immunologic memory by stimulations of various DAMPs/PAMPs such as lysophosphatidylcholine (LPC) (17). Shao et al. paper further reported that the expressions of 1311 innate immune regulators are modulated in 21 human endothelial cell transcriptomic datasets by various DAMPs/PAMPs including Middle East Respiratory Syndrome Coronavirus (COVID-19 homologous virus), lipopolysaccharide (LPS), LPC, shear stress, hyperlipidemia and oxidized low-density lipoprotein (oxLDL) (Shao et al.). In addition, another major cell type in the aortic wall, VSMCs can also undergo metabolic reprogramming to build this immunologic memory by oxidized low-density lipoprotein (oxLDL) stimulation (Schnack et al.). Last but not least, it was a well-documented concept that monocytes and macrophages as prototypic innate immune cell types can establish trained immunity (38). Recent studies further confirmed that the macrophages can be polarized into pro-inflammatory macrophages (M1), anti-inflammatory macrophages (M2), tumor-associated macrophages, adipose tissue macrophages and many other macrophage subsets (Lai et al.) in different conditions (Zhang et al.). The LPS/interferon-γ or SARS-CoV-2 Coronavirus spike protein treatment can push pro-inflammatory macrophage (M1) formation and upregulate ATP-citrate lyase (ACLY), which is the key enzymes catalyzing metabolic reprogramming. The M1 macrophages then switch their metabolism from oxidative phosphorylation (OXPHOS) to glycolysis (Barhoumi et al.; Dominguez et al.). Finally, in a recent paper found after transcriptomic and epigenetic rewiring of granulopoiesis, the trained granulopoiesis promotes an anti-tumor immune phenotype in neutrophils (39). In the inflammatory conditions, the secretion of neutrophil extracellular traps can further stimulate neutrophils to amplify inflammatory response (Domer et al.; Perez-Figueroa et al.). All the evidences indicate that aorta can build the immunologic memory and amplify the immunologic responses in pathological conditions.

## A new concept: Aorta may serve as an immune organ in pathologies

Because of the ability to provide a niche for immune cell maturation, differentiation, and activation, lymph nodes and spleen were defined as a peripheral immune organ (40). Current understanding in this field indicated that the aorta may have similar abilities to provide a microenvironment/niche for endothelial cell phenotype switch to mesenchymal cells (4, 16, 41), vascular smooth muscle cells (VSMCs) phenotype switch to macrophages (21, 42) and other five different plastic cell types (43), monocyte differentiation (26, 44, 45), macrophage differentiation (Zhang et al.; 46, 47), neutrophil activation (Domer et al.) as well as T cell and B cell differentiation (44, 48, 49). Based on the principle the same as immunologists previously defined lymph nodes and spleen as immune organs, in terms of function, the aorta can serve as an immune organ in pathological conditions. However, the key knowledge gaps are a) how immune cell differentiation, trans-differentiation, activation take place in aorta and what are the molecular mechanisms underlying the processes. A recent paper found the secretomes (18, 50) (Ni et al.) in aorta play a significant role in providing the microenvironment for immune cell maturation, differentiation and activation. In this paper, author found that approximately 53.7% out of 21,306 human protein-encoding genes are classified into six secretomes including canonical secretome (secretory proteins with signal peptide), caspase 1-GSDMD secretome, caspase 4-GSDMD secretome, exosome secretomes, Weibel–Palade bodies, and autophagy secretome. Those six types of secretomes were significantly modulated in the aorta of pathological condition, including aorta in atherosclerosis, chronic kidney disease, abdominal aortic aneurysm or endothelial cell treated with Middle East Respiratory Syndrome Coronavirus, vascular smooth muscle cell treated with Angiotensin II (Lu et al.). This finding not only filled the knowledge gap by providing us a novel insight that aorta may serve as an immune organ and regulate the immunological response *via* six types of secretomes in pathological conditions, but also raise the potential targets for future therapeutic interventions for inflammation, cardiovascular disease and autoimmune disease.

In this editorial, we summarized five significant papers (Ellen et al.; Lu et al.; Stolarz et al.; Xu et al.) published on our Research Topic in Frontiers in Immunology, Molecular Innate Immunity Section to illustrate the most recent understanding on molecular innate immunity.

## Author contributions

YL and YuS carried out literature collections, research analyses, and drafted the manuscript, KX, YiS, FS, NS, LY, JY, SW, WH, JS, and HW provided editing input. XY supervised and edited the manuscript. All authors listed have made a substantial, direct, and intellectual contribution to the work and approved it for publication.

## Funding

This work was supported by the National Institutes of Health Grants to XY (HL132399-01A1; HL138749-01; and HL147565-01), HW (DK104116; DK113775; and HL131460)

## Conflict of interest

The authors declare that the research was conducted in the absence of any commercial or financial relationships that could be construed as a potential conflict of interest.

## Publisher’s note

All claims expressed in this article are solely those of the authors and do not necessarily represent those of their affiliated organizations, or those of the publisher, the editors and the reviewers. Any product that may be evaluated in this article, or claim that may be made by its manufacturer, is not guaranteed or endorsed by the publisher.
